# Heat therapy for different knee diseases: expert opinion

**DOI:** 10.3389/fresc.2024.1390416

**Published:** 2024-07-04

**Authors:** Roberto Rossi

**Affiliations:** ^1^ Surgical Department, Mauriziano Umberto I Hospital, Turin, Italy; ^2^ Department of Surgical Sciences, University of Turin, Turin, Italy

**Keywords:** knee, pain, thermal therapy, heat, cold, osteoarthritis, rheumatoid arthritis

## Abstract

Musculoskeletal pain is a major burden in our society. Management of musculoskeletal pain or injuries includes both pharmacological and non-pharmacological approaches, including heat therapy (HT). HT is a well-established treatment option due to its ability to promote muscle relaxation, enhance blood circulation, and modulate nociceptors with a good safety profile. The main focus of this paper is to review the available literature about HT in knee pathologies (i.e., arthrosis, arthritis, traumatic pathologies in the subacute phase, muscle and tendon pathologies linked to fatigue, muscle tension and distractions) and to provide an expert opinion in case of lack of data.

## 1 Introduction: heat therapy and cold therapy in orthopedics

Musculoskeletal pain is a major burden in our society, being associated with significantly impaired functional ability, reduced quality of life, emotional distress, and sleeping disorders. Studies consistently report that the most affected areas are knee, back and neck (1–3). Despite its high prevalence, musculoskeletal pain is still undertreated: a European telephone survey demonstrated that one in four persons generally do not ask for medical treatment of pain (4). This is of pivotal interest, since adequate cure of acute pain prevent its evolution in chronic pain, with severe consequences (5).

Management of musculoskeletal pain or injuries includes both pharmacological and non-pharmacological approaches. The first are the generally accepted first-line treatment, leading to abuse due to their widespread availability (6). Among the latter, physical exercise and thermal therapy encompass a growing role. Thermal therapy is defined as a non-pharmacological therapeutic application of any intervention to the body that increases or reduces heat, namely heat therapy (HT) and cold therapy (CT), resulting in elevating or reducing tissue temperature, respectively. Recent guidelines recommend the implementation of preventative strategies and physical tools as the first option to minimize the use of medications. In patients who have had an inadequate response to drugs, the proper use of other alternative techniques is vital for safe and effective management of chronic pain patients (6). In particular, the most recent guidelines of the American College of Rheumatology about the management of osteoarthritis of the hand, hip, and knee consider non-pharmacological treatments as effective options, including HT and CT (7), in order to facilitate a return to normal function and activity (8).

**Figure 1:**
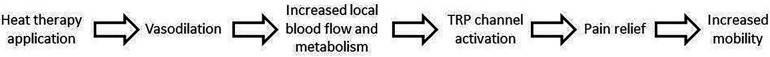
Pathophysiological effects of heat therapy.

HT can be delivered either superficially or deeply. Superficial HT can be delivered by conduction (wearable heat wraps, heat packs with grain, hot water bottles, hot poultices, hot stones, electric heat pads) or convection (hydrotherapy, hot baths, heat lamp, stream/sauna). On the other hand, deep HT is delivered by conversion (ultrasound, diathermy, laser therapy) (8–12). It acts on musculoskeletal pain in multiple ways. The application of low-level superficial heat activates temperature-sensitive nerve endings (thermoreceptors), which, in turn, initiate signals that block the processing of pain signals (nociception) in the lumbar dorsal fascia and spinal cord (13). In addition, the pressure used to apply some superficial heat therapies, such as heat wraps, may activate the nerve endings that detect changes in tissue pressure and movement (proprioceptors); when activated, the proprioceptors block the transmission of pain signals to the spinal cord and the brain. The analgesic effects of heat are partly mediated by TRPV1 receptors, which facilitate the neural transduction of heat and the processing of nociceptive pain. The activation of TRVP1 receptors in the brain is thought to regulate anti-nociceptive pathways. These mechanisms serve to reduce muscle tonicity and relax muscles, thereby reducing spasms and musculoskeletal and increasing muscle flexibility (8, 9, 14). Figure 1 resumes the mechanisms described above.

In addition, an increase in temperature tends to reduce the stiffness in fascial tissues (15). Moreover, it can also lead to increased metabolism and vasodilation, thus accelerating the healing processes by enhanced supply of nutrients and oxygen and removal of pain-inducing mediators produced as a by-product of tissue damage. An elevation in tissue temperature of just 1°C is associated with a 10%–15% increase in the local metabolism. Connective tissues may also change in viscosity and density in response to heat, thereby improving the range of movement and enhancing tissue extensibility. Recent evidence also suggests that localized, repeated HT may promote an angiogenic environment and enhance muscle strength (8–10, 16, 17). Potential risks to use HT are burns and skin ulceration, as well as, in some cases, the exacerbation or the progression of inflammation. Contraindications to HT can be resumed as heat hypersensitivity, burn areas, open or healing wounds or skin damage, undiagnosed acute musculoskeletal injuries, inflammatory processes, actual or suspected bacterial infective or rheumatologic diseases in active phase (8, 9).

CT or cryotherapy, instead, is part of the RICE protocol, namely “rest, ice, compression and elevation”, which is often used in the acute phase of musculoskeletal injuries (9, 18). In contrast to HT, CT reduces blood flow to the cooled tissues, thus leading to vasoconstriction via a sympathetic reflex (8, 9). Decreasing blood flow implies reduction of edema and slower delivery of inflammatory mediators to the injured area, meaning reduced inflammation (19, 20). Interestingly, the decreased metabolic demand in the cooled zone also prevents secondary hypoxia-related damage (21, 22). In addition, CT produces local anesthesia, namely cold-induced neurapraxia, by decreasing the activation threshold of tissue nociceptors and the conduction velocity of nerve signals conveying pain (9, 23). Finally, decreasing muscle temperature also reduces muscle spasm via inhibition of a spinal cord reflex loop (24). Complications of wrongly applied CT are frostbite, allergic reactions, burns, intolerance or pain, temporary nerve palsy (25, 26). Contraindications to CT are cold hypersensitivity, cold intolerance, Raynaud's disease, or areas of vascular compromise (8).

## 2 Indications to heat therapy in musculoskeletal pathologies

The so-called superficial HT represents an effective, safe and inexpensive treatment applicable in many musculoskeletal pathologies in different contexts, namely hospital, rehabilitation clinics and at home (27). Firstly, HT has been shown to have benefits in the treatment of arthrosic (or osteoarthritic) pain and related muscle stiffness (28). In particular, the circulatory vasodilatory effect, thanks to the greater blood support and oxygenation to damaged cells, makes HT typically useful in the subacute or chronic phases of the injury. However, to date the main applications of superficial HT are non-specific muscle (axial ore extremities) pain or joint pain, associated for example with excessive strain, as well as strains, sprains and arthritis (8, 29–33). Low back pain is a typical indication for HT, after excluding systemic causes (34, 35). In a review including a total of 1,117 adults patients, French et al. indicated that the continuous application of low-level heat directly to the skin via a heat wrap was shown to provide small, short-term improvements in pain and mobility (36). Interestingly, in two workplace studies in adults, heat wrap therapy was found to significantly reduce pain intensity in patients with acute low back pain, both during treatment and up to two weeks after its use (37, 38). Heat wrap therapy also reduced the impact of pain on everyday activities, most notably the ability to lift, work performance, and quality of sleep, and provided sufficient pain relief for most patients during treatment and two weeks after its use (37). Generally, a multimodal approach including manual therapy, therapeutic exercise, thermal therapies (such as HT and CT) and proper information and self-management tactics are crucial in the management of these conditions, in order to avoid drug overuse and to favor recovery. Indeed, the combination of HT with exercise in adults was associated with significantly greater short-term pain relief when compared to with the exercise alone and no treatment/placebo control groups (39). In the chronic setting, heat wrap combined with basic multimodal treatment determined greater improvements in their parameters (extension and right/left rotation) after 12 weeks in adults (10), as well as short-term improvements in physical and psychological wellbeing (40). Finally, particular settings that may benefit from HT are: pre-competition preparation to warm up muscles, to soften tissues in case of stiffness due to post-traumatic immobilization, and to reduce pain and warm up a tendon affected by tendinopathy; and delayed-onset muscle soreness (DOMS) (30, 41–44). The main focus of this paper is to review the available literature about HT in knee pathologies and to provide an expert opinion in case of lack of data. Table 1 resumes the indications of HT and CT in the different knee pathologies, which are analyzed in detail below.

**Table 1:** Indications of heat therapy and cold therapy in knee pathologies.

Pathology	Heat therapy	Cold therapy
Knee ostheoarthritis (arthrosis)	In the subacute and chronic phases to improve quality of life, pain, stiffness, functional capacity and general health perception. Before exercise to increase compliance and healing, favoring pain recovery	In the acute phases to reduce blood flow and favor vasoconstriction
Knee arthritis/inflammatory disease	In the chronic phases to improve symptoms and joint function	In the acute phases to reduce inflammation
Traumatic pathologies (sprains, meniscal tear)	In the subacute phase (after 7 days) to reduce pain	In the acute phase
Muscle and tendon pathologies linked to fatigue, muscle tension and distractions (tendinitis, tenosynovitis, strains)	In the acute and subacute phase, combined with cold therapy. In the chronic phase alone	In the acute and subacute phase, combined with heat therapy

## 3 Focus on knee and heat therapy

### 3.1 Degenerative knee pathology (arthrosis)

Arthrosis or osteoarthritis is a degenerative disease linked to wear and tear on the joints, which mainly affects people over the age of 50. Increasing global lifespan has in turn increased the prevalence of arthrosis, which is now the most common type of arthritis (45). One tenth of the world's population suffers from an arthritic pathology at some point in their life, with a higher prevalence among women. In 2017, arthrosis was found to have affected approximately 300 million people globally, predominantly in the hip and knee joints (46). In arthrosis, there is a progressive degeneration of the cartilage of a single joint and a hypertrophy of the underlying bone, which very often leads to new bone formation in the form of osteophytes which rub together, causing persistent pain and limitation of movement. Arthrosis is defined as primary if caused by a primary metabolic alteration of the articular cartilage, secondary when linked to trauma or iatrogenic causes. The clinical manifestations of arthrosis are characterized by pain, inflammation of the joints with hot and red skin, stiffness and reduction in range of motion, which is often associated with weakness and loss of muscle tone at the affected joint, enlarged deformity of the articulation. Other symptoms are the presence of cracking at the joint level and the patient's sensation of instability. This is a long-lasting symptomatology: arthrosic pain generally lasts for many years and worsens with use of the joint; it is often asymmetrical pain, for example between the two knees, the two wrists, the two hands. Arthrosis mainly affects the spinal column (cervical and lumbar); knees and hips (weight-bearing joints); the hands and in particular the thumb, with pain that arises for example when the patient holds an object. Risk factors for osteoarthritis include weight, family history (with a greater likelihood of developing the disease if parents are affected), age and female sex. Today it is not yet possible to cure arthrosis, what can be done is to intervene on the pain by trying to control it and encourage a correct lifestyle, with control of body weight, while in advanced cases the prosthesis is the indicated treatment.

In the case of arthrosis, the weight-bearing joints are the most affected. In particular, the knee appears to be the joint most subject to the development of arthrosis as it is a joint that supports a significant weight and is subjected to the effects of obesity, trauma and some metabolic diseases. However, the main cause of knee arthrosis is advancing age: in fact, after the age of 60, it is common to experience severe trauma to the knee, systemic diseases, metabolic diseases and microtraumas repeated over time. Since there is currently no cure for knee arthrosis, the therapeutic approach is based on the management of painful symptoms, maintenance or improvement of mobility and minimization of disability, to be achieved both with pharmacological intervention and non-pharmacological therapies such as exercise and HT. Indeed, international guidelines recommend thermal agents among non-pharmacological treatments of knee osteoarthritis (47). In studies conducted on patients suffering from knee osteoarthritis, the application of HT significantly improved quality of life, pain, stiffness, functional capacity and general health perception (28, 48–51). In addition, HT application before home exercise programs can also increase compliance and healing, favoring pain recovery (30). Indeed, the use of superficial HT increases blood supply and stimulates connective and tissue regeneration in a physiological way, as indicated by knee intracapsular, intramuscular and skin temperature elevation (52). More in detail, in the knee joints, the tissue temperature is approximately 30°C, 7°C lower than that of the core. Considering that tissue metabolism doubles approximately every 3°C increase in temperature, the application of superficial heat can quadruple skin metabolism with an important contribution to repair mechanisms (14). Moreover, HT combined with exercise seems to be more effective in ameliorating symptoms than exercise alone in knee osteoarthritis; this is particularly the case of Spa therapy together with physical exercise (53–60). Notably, in some cases it is necessary to combine HT with CT to increase the vascular stimulus (vasodilator and vasoconstrictor effect, respectively) (61). In fact, while HT is useful in the subacute or chronic phases, in the acute phases with swelling and/or hematoma with often inflammatory vasodilation the use of CT is necessary, to reduce blood flow and favor vasoconstriction.

### 3.2 Inflammatory pathologies (arthritis)

The most common and widespread forms of knee arthritis are rheumatoid arthritis, gouty arthritis, or forms associated with rheumatologic diseases such as psoriasis or systemic lupus erythematosus. These are chronic inflammatory pathologies of autoimmune origin which can occur at any age and are characterized by pain and inflammation, at the level of both large and small joints. The pain linked to arthritis, unlike arthrosic pain, worsens at rest and the stiffness, typically in the morning and prolonged, represents a sign of a disease that affects the entire organism. Among the causes of arthritis, which are not yet completely clear, there are genetic, environmental and lifestyle factors (62). Some forms are characteristic of the female sex, therefore gender (due to hormonal production) also seems to be involved.

Nearly 90% of patients suffering from rheumatoid arthritis have involvement of the knee joint, typically symmetrically. Stiffness lasts >60 min after rising in the morning but may occur after any prolonged inactivity (called gelling). Involved joints become tender, with erythema, warmth, swelling, and limitation of motion. The spectrum of knee involvement can range from chronic synovitis without significant bone destruction to complete loss of cartilage with severe bone loss and severe soft tissue contractures (63). Diagnosis is based on specific clinical, laboratory, and imaging features. Clinically, up to 30% patients present subcutaneous rheumatoid nodules. Other extra-articular signs include vasculitis causing leg ulcers, digital ischemia, or multiple mononeuropathy (mononeuritis multiplex), pleural or pericardial effusions, obliterative bronchiolitis, interstitial lung disease, pericarditis, myocarditis, lymphadenopathy, Felty syndrome, Sjögren syndrome, scleromalacia, and episcleritis. At blood tests, 80% of patients suffering from rheumatoid arthritis have rheumatoid factor and 100% have antibodies against citrullinated proteins (ACPA/anti-CCP).

Treatment of arthritis patients focuses on relief of symptoms and improvement of joint function. Generally, it involves the use of several strategies, or combinations of treatments. Physical therapy can often be combined with drug therapy as exercises can improve the range of motion and strengthen the muscles surrounding the joints. Regarding drugs, cortisone, methotrexate, biologic drugs, or small molecules can be used depending from the disease (64, 65).

The use of topical HT in individuals with rheumatoid arthritis in chronic phase must be carefully evaluated depending on the phase of the disease. In clinical practice, when a joint is reactive and swollen, HT is not indicated, while in the chronic phases when the joint is painful without signs of inflammation, namely swelling, HT can be considered to improve symptoms. In addition, HT added to a multimodal rehabilitative treatment including exercises, physiotherapy, occupational therapy and galvanic baths have demonstrated beneficial long-term effects with long-lasting improvements in pain intensity, as well as reduced consumption of corticosteroids and NSAIDs and/or analgesics in patients with rheumatoid arthritis (66).

### 3.3 Traumatic pathologies in the subacute phase

Sprains are defined as tears in ligaments and can be classified into: 1st degree or minimal (fibres are stretched but intact, or only a few fibres are torn), 2nd degree or partial (some to almost all fibres are torn), and 3rd degree or complete (all fibres are torn). Anterior Cruciate Ligament Sprain is the injury to the anterior cruciate ligament which usually occurs because of noncontact deceleration forces, as when a runner plants one foot and sharply turns in the opposite direction. The patient usually reports hearing or feeling a “pop” at the time of the injury, and must cease activity or competition immediately. Swelling of the knee within two hours after the injury indicates rupture of the ligament and consequent hemarthrosis (67). Medial Collateral Ligament Sprain, instead, is the injury to the medial collateral ligament is fairly common and is usually the result of acute trauma. The patient reports a misstep or collision that places valgus stress on the knee, followed by immediate onset of pain and swelling at the medial aspect of the knee (67). Lateral Collateral Ligament Sprain, finally, is the injury of the lateral collateral ligament is much less common than injury of the medial collateral ligament. Lateral collateral ligament sprain usually results from varus stress to the knee, as occurs when a runner plants one foot and then turns toward the ipsilateral knee. The patient reports acute onset of lateral knee pain that requires prompt cessation of activity (67). The meniscus can be torn (meniscal tear) acutely with a sudden twisting injury of the knee, such as may occur when a runner suddenly changes direction. Meniscal tear also may occur in association with a prolonged degenerative process, particularly in a patient with an anterior cruciate ligament–deficient knee. The patient usually reports recurrent knee pain and episodes of catching or locking of the knee joint, especially with squatting or twisting of the knee (67, 68).

Globally, knee sprains are acute traumatic injuries, necessitating of CT in the acute phase. Many partial tears heal spontaneously, while complete tears often require surgery to restore anatomy and function. Indeed, after the first 7 days, in partial tears HT can be considered to reduce pain, as can be derived by a meta-analysis of heterogeneous studies (51).

### 3.4 Muscle and tendon pathologies linked to fatigue, muscle tension and distractions

These muscle and tendon pathologies are often linked to physical effort, running, sprints and jumps and can concern athletes and non-athletes. Such muscle and tendon inflammation is often linked to overload or excessive activity which can cause chronic inflammation over time (69–71). Tendinitis is defined as the inflammation of a tendon, often developing after degeneration (tendinopathy), while tenosynovitis is tendinitis with inflammation of the tendon sheath lining. Symptoms usually include pain with motion and tenderness with palpation. Chronic deterioration or inflammation of the tendon or tendon sheath can cause scars that restrict motion. Diagnosis is clinical, sometimes supplemented with imaging. In case of knee, the most common disease of such kind are patellar, quadriceps and quill leg tendinopathies. Generally, treatment includes rest, anti-inflammatory drugs, and sometimes corticosteroid injections. However, in the acute and subacute phase, combination of HT with CT can be considered, while in the chronic phase HT only can have a role.

Strains are defined as tears in muscles and can be graded as sprains. In the knee context musculotendinous distraction can regard the vastus medialis oblique, the rectus femoris, the vastus lateralis, the twin muscles (posterior to the knee), the biceps femoris and semimebranosus muscles. In case of distraction (1st grade) or partial tears (2nd grade), healing is spontaneous. Also in these cases, in the acute and subacute phase combination of HT with CT can be considered, while in the chronic phase HT only can have a role.

## 4 Gaps in knowledge, future directions and conclusions

Nowadays it is essential to improve the patient’s quality of life, reducing pain as much as possible. The cause of persistent pain often leads to worsening of cognition (such as depression) and relationships. Often the patient reduces daily movement to avoid having a painful joint, often compromising other comorbidities that require daily movement (for example diabetic subjects or those with vascular and cardiac pathologies). The use of HT for knee pathologies allows to reduce pain, increase blood supply to the injured tissue and stimulate connective and tissue regeneration by exploiting the action of heat (Table 1). Several fields of application can be explored, so that HT can have a role as a complementary, easy-to-use and inexpensive treatment in different knee pathologies.
